# Considering ecological traits of fishes to understand microplastic ingestion across Pacific coastal fisheries

**DOI:** 10.1371/journal.pone.0339852

**Published:** 2026-01-28

**Authors:** Jasha Dehm, Kelly Thomas Brown, Eseta Drova, Rufino Varea, Joycinette Vosumbe Botleng, Siutiti Fe’ao, Lavata Nivaga, Laura Williams, Brian L. Stockwell, Salanieta Kitolelei, Cherie Morris, Nanise Kuridrani, June Brian Molitaviti, Vailala Matoto, Lotokufaki Paka Kaitua, Semese Alefaio, Hudson Feremaito, Krishna Kumar Kotra, Amanda Kirsty Ford

**Affiliations:** 1 Centre for Sustainable Futures, The University of the South Pacific, Suva, Fiji; 2 Pacific Islands Climate Action Network, Suva, Fiji; 3 Graduate School of Bioresource and Bioenvironmental Sciences, Kyushu University, Fukuoka, Japan; 4 Leibniz Centre for Tropical Marine Research (ZMT), Bremen, Germany; 5 Ministry of Fisheries and Forestry, The Government of Fiji, Suva, Fiji; 6 Vanuatu Fisheries Department, Ministry of Fisheries, Oceans and Maritime Affairs (MFOMA), The Government of Vanuatu, Port Vila, Vanuatu; 7 Ministry of Fisheries, The Government of Tonga, Nuku’alofa, Kingdom of Tonga; 8 Tuvalu Fisheries Department, The Government of Tuvalu, Funafuti, Tuvalu; 9 School of Agriculture, Geography, Environment, Ocean and Natural Sciences, The University of the South Pacific, Emalus Campus, Port Vila, Vanuatu; VIT University, INDIA

## Abstract

Coastal fisheries are essential to Pacific Island communities, providing vital nutrition, livelihoods, and cultural value, yet microplastic (MP) contamination poses a growing threat to both ecosystem and human health. This study presents a regional assessment of microplastic contamination in coastal fish across four Pacific Island Countries and Territories (Fiji, Tonga, Tuvalu, and Vanuatu), based on the compilation of four methodologically standardized datasets, enabling us to evaluate whether regional patterns of contamination are linked to the ecological traits of fish. A total of 878 fish from 138 species were analysed to reveal widespread ingestion (32.7% prevalence; 0.76 ± 0.05 MPs/individual), with Fiji exhibiting the highest contamination (74.5% frequency). Reef-associated invertivores such as *Lethrinus harak* showed elevated risks (80% contaminated in Fiji), driven by fiber-dominated particles (65–95%), while ecological traits (benthic feeding, reef habitats) increased exposure compared to nearshore pelagic species. Disparities emerged between nations, with Fiji’s sites exceeding global averages despite remoteness, whereas Vanuatu’s low fish contamination suggests restricted dispersal, successful waste management influences, or differential bioaccumulation pathways. Polypropylene, polyethylene, polyethylene terephthalate, and nylon were the dominant (~11–43%) polymer types across all countries. The findings highlight the essential need to incorporate the Pacific Island data into global pollution assessments to better represent tropical Pacific marine ecosystems. This work establishes a standardized baseline for microplastics in the Pacific coastal fish, providing a framework to guide future research on ecological impacts while highlighting the need to integrate these data into regional and global plastics negotiations. The study underscores the importance of expanding monitoring to underrepresented PICTs to better understand contamination drivers in island ecosystems.

## Introduction

The accumulation of plastic debris in marine environments has become a pressing global concern, with microplastics (plastic particles smaller than 5 mm) emerging as a pervasive and persistent environmental containment [1–3]. These particles originate either as intentionally manufactured small particles, known as primary microplastics, or through the fragmentation of larger plastic items, termed secondary microplastics [4,5]. Once introduced into marine ecosystems, microplastics are transported across vast distances, contaminating diverse habitats ranging from surface waters and coastal sediments to the deep-sea floor [6,7]. Their ubiquitous presence has raised significant concerns about their potential impacts on marine organisms and ecosystems, with ingestion documented in a wide array of marine species, including fish, invertebrates, and even large filter-feeders such as whales [3,8,9].

Despite significant global efforts to understand microplastic pollution, there remains a critical gap in knowledge regarding its presence and distribution in the Pacific Island Countries and Territories (PICTs) [10,11]. While a handful of independent studies have been conducted, such as those in Fiji, French Polynesia, Solomon Islands, Tonga, and Vanuatu, these investigations lack regional coordination, standardized methodologies, and standardized reporting (e.g., units), preventing meaningful cross-country comparisons. For instance, reported microplastic concentrations in fish vary widely, from 0.86 to 4.7 MPs/fish for the same year and island (2021, Viti Levu in Fiji), with similar inconsistencies in water and sediment samples (Table 1). Furthermore, global reviews and meta-analyses, such as those by Sequeira et al. [12] and Sacco et al. [13], largely exclude PICTs data, focusing instead on the Global North, including China, the U.S., and Europe. This omission has led to regional assumptions being based on sparse, uncoordinated studies, with no comprehensive baseline to inform policy or scientific hypotheses.

**Table 1:** Overview of marine microplastic research within the Pacific Island Countries and Territories. Market surveys marked by asterisk (*); otherwise, data reported from field surveys. FO = Frequency Occurrence.

Country	Year	Sample type	FO (%)	Concentration	References
Fiji	2020	Coastal surface water	–	1.6 (rural) – 2.0 (urban) MP/L	[14]
Fiji	2020	Sediment	81.8	0.008–0.034 MPs/g	[15]
		Coastal surface water	94.4	0.09–0.24 MPs/m^3^	
		Coastal fish (five families)	68	5.5 ± 9.4 MP/fish	
Fiji	2022	*Batissa violacea*	100	2.78–6.84 MP/g dry weight	[16]
Fiji*	2021	Coastal fish (four species)	35.3	0.86 ± 0.14 MP/fish	[17]
Fiji	2021	*Lethrinus harak*	100	4.7 ± 0.9 (wet season), 3.3 ± 1.3 (dry season) MP/fish	[18]
Fiji	2022	Coastal surface water (Seabin)	–	0.03 MP/m^3^	[19]
Fiji	2022	Beach sediment	–	4.5 ± 11.1 MP/m^2^	[20]
Fiji	2024	*Anadara antiquata*	64	5.93 ± 0.39 MP/individual	[21]
Fiji	2025	Wild oyster	6	1.78 ± 1.04 microplastics/100 g	[22]
		Farmed oyster (*Saccostrea mordax*)		0.80 ± 0.20 MPs/L	
		Coastal surface Water			
Fiji	2025	*Anadara* spp. (1980s)	76	0.42 ± 0.4 MP/individual	[23]
		*Anadara* spp. (2023-2024)	100	0.93 ± 0.4 MP/individual	
Fiji	2025	Sediment (mangroves)	-	13.79 ± 1.24 N kg−1 dw	[24]
		Sediment (adjacent to mangroves)	-	1.42 ± 0.26 N kg−1 dw	
		Sediment (seaward to mangroves)	-	2.14 ± 0.40 N kg−1 dw]	
French Polynesia	2017	Coastal surface water	–	0.74 MP/m^2^	[25]
French Polynesia	2019	Coastal fish (four genera)	21	1.25 MP/fish	[26]
Solomon Islands	2020	Sediments	–	450–15,167 MPs/Kg	[27]
Tonga	2022	Surface Water	–	1.05 ± 0.13 MP/m^3^	[28]
Tonga	2023	Intertidal sediment	–	23.5 ± 1.9 MP/L sediment	[29]
		Subtidal Sediments	–	15.0 ± 1.9 MP/L sediment	
Vanuatu	2020	Sediments	–	333–33,300 MP/kg	[27]
		Coastal surface water	–	0.09–0.57 MP/m^3^	
		Crabs	57	1.71 ± 2.27 MP/crab	
		Reef fish (four families)	35	2.9 ± 4.6 MP/fish	
		Tuna	83	4.3 ± 5.13 MP/fish	

The urgency of addressing these gaps is heightened by the global environmental threat posed by microplastics, which persist across ecosystems, transport widely, and contribute to the transgression of planetary boundaries [30,31]. The ‘novel entities’ boundary, which includes microplastics, is already considered well beyond safe limits, yet quantification remains uncertain, particularly in data-poor regions like the PICTs [31,32]. Without regional data, effective policy development and management strategies are hindered, leaving PICTs disproportionately vulnerable [32]. Moreover, plastic production and degradation exacerbate all other planetary boundaries [33], further underscoring the need to fill knowledge gaps. A unified regional assessment is therefore essential not only for local decision-making but also for understanding the broader ecological and planetary impacts of microplastic pollution.

Fish, as integral components of marine food webs, play a critical role in both ecosystem functioning and human livelihoods [34–36]. In many parts of the world, particularly in the PICTs, fish are a vital source of protein and essential nutrients, underpinning food security and cultural practices [37,38]. However, fish are also recognized as potential vectors for the transfer of impurities, including microplastics, from the environment to humans [13,39]. Studies have shown that microplastic ingestion in marine fish is widespread, with evidence of contamination found in species across various trophic levels and habitats [13,40]. Microplastic ingestion thus raises concerns not only for the health of marine organisms but also for human consumers, as plastic may leach harmful additives or act as carriers for pathogens and toxic chemicals, such as persistent organic pollutants and heavy metals [39,41]. Despite these risks, research on microplastic contamination in fish, particularly in species consumed by humans in PICTs, remains limited.

Assessing microplastic exposure in fishes using ecological traits provides an interesting framework for understanding microplastic contamination pathways. Such an approach, which categorizes species according to feeding mode (e.g., herbivores, planktivores, invertivores, piscivores), feeding strategy (e.g., suction feeders, ram feeders), habitat use (e.g., benthic demersal, reef associated) and feeding zone (e.g., benthic, pelagic), helps predict exposure pathways since these groupings reflect distinct niches and foraging behaviors. For example, planktivorous fishes may be particularly susceptible to ingesting microplastics due in part to their filter-feeding behavior and potential to mistake plastic particles for zooplankton [42,43]. Predatory species at higher trophic levels may accumulate microplastics through trophic transfer from contaminated prey [44]. Benthic feeders may ingest (unintentionally) microplastics directly from sediments, while herbivores may consume plastics adhered to algae. Thus, by integrating these ecological traits into analysis, researchers can better identify the mechanisms and variability in microplastic exposure across diverse fish assemblages.

PICTs are uniquely vulnerable to the impacts of marine plastic pollution. High urbanization rates, heavy reliance on coastal fisheries, and limited waste and water management systems exacerbate the risk of microplastic contamination in these regions [45]. Inadequate waste management infrastructure [46], coupled with a lack of water treatment facilities, allows plastic debris to enter marine ecosystems more readily, where it degrades into microplastics [47]. These challenges are compounded by the region’s dependence on marine resources for subsistence and economic stability [48]. Despite these vulnerabilities, there has been little research comparing microplastic contamination across different PICTs, and existing studies often employ differing methodologies, making regional comparisons difficult. Furthermore, much of the research on fish in PICTs has focused on species supplied to urban markets or targeted by commercial fisheries [17], rather than those caught and consumed by coastal communities for subsistence. This gap in knowledge limits our understanding of microplastic contamination in subsistence fish that are integral to the diets and food security of local populations.

In this study, we aim to address key research gaps by investigating the frequency of occurrence and abundance of microplastics in the gastrointestinal tracts of important coastal fish species from four PICTs: Fiji, Vanuatu, Tonga, and Tuvalu. Using an opportunistic sampling approach of landed fish within communities, we ensure that the fish sampled are representative of those consumed for subsistence. Our objectives are twofold: (a) to compare microplastic contamination across commonly caught fish species to identify potential regional similarities and differences in contamination levels and (b) to evaluate the role of ecological traits (including feeding type, feeding strategy, habitat, feeding zone) in influencing microplastic contamination and identify which traits are associated with higher contamination risks.

The findings of this study contributes to a broader understanding of microplastic pollution in a data-limited region, with direct implications for food security, public health, and waste management. By examining commonalities in microplastic contamination across PICTs and evaluating the influence of ecological traits, we identify ecological patterns and potential drivers of contamination. These insights help pinpoint fish groups that may serve as effective bioindicators and highlight priorities for further research. The findings also underscore the need for targeted, region-specific strategies to address plastic pollution in the Pacific, where the environmental and human health risks are particularly acute.

## Method

The study used biodiversity datasets published for Fiji [49], Tonga [50], Tuvalu [51], and Vanuatu [52] via the Global Information Biodiversity Facility (www.gbif.org). These datasets were compiled under the Asia Pacific Network project “*Establishing Baselines for Marine Plastics and Bridging Indigenous Knowledge with Ocean Policy to Improve Livelihood Security in the Pacific*” and included quantifying microplastics in coastal fish.

**Fig 1:**
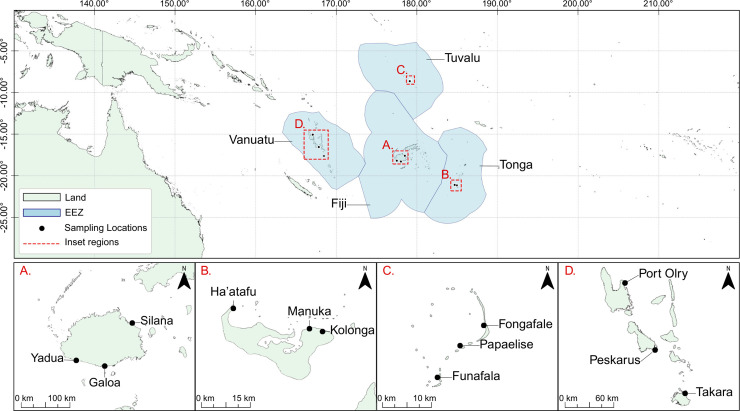
Sample origins from coastal communities in Fiji, Tonga, Tuvalu, and Vanuatu. (A) Galoa Village, Silana Village, and Yadua Village (Viti Levu, Fiji). (B) Ha’atafu Village, Manuka Village, and Kolonga Village (Tongatapu, Tonga). (C) Fongafale Islet, Papaelise Islet, and Funafale Islet (Funafuti Atoll, Tuvalu). (D) Takara A Village (marked as “Takara” on map), Mele Fisher Community, Ifira Fishing Community (Efate Island), Peskarus (Maskelyne Island/ Malekula), and Port Olry (Espiritu Santo, Vanuatu). Countries shapefile (CC-BY) was obtained from www.naturalearthdata.com and Exclusive Economic Zone shapefile (CC-BY) is obtained from www.marineregions.org.

Detailed stie descriptions and sampling methodology are described in detail in Drova et al. [53] for Fiji and Fe’ao et al. (Tonga, [54]), Alefaio et al. (Tuvalu), and Botleng et al. (Vanuatu, [55]), which are in review. The full dataset is comprised of fish collected from a total of 14 fishing communities (Fig 1) from across Tongatapu (Tonga), Funafuti (Tuvalu), Viti Levu (Fiji), and from Espiritu Santo, Malekula, and Efate (Vanuatu).All fish specimens were obtained post-mortem from local fishers and were not killed or handled alive by the research team, hence in accordance with national regulations and the University of the South Pacific’s requirements, animal ethics approval from university ethics committee was not required for this study. National permits were obtained from the relevant authorities in each country: Fiji (MTA-42/2–3, Ministry of iTaukei Affairs), Tonga (ORG 1/8 v.24, Ministry of Education and Training), Tuvalu (approval from the Director of the Tuvalu Fisheries Authority), and Vanuatu (VAN-ENV-04524).

The process of microplastic extraction, visual analysis and polymer identification were kept consistent across all four countries, and followed established methodologies described by Foekema et al. [56], Ferreira et al. [15], and Wootton et al. [17]. In summary, gastrointestinal (GI) tracts were digested using 30% H₂O₂, processed using a sieve series and visually analysed via microscopy. Quality assurance and quality control measures were maintained across all studies, including sterile conditions, contamination controls, and validation of a subset of identified microplastics using Fourier Transform Infrared Spectroscopy (FTIR) polymer analysis following methods described by Jung et al. [57].

Following the merging of the four GBIF datasets, ecological traits (Table 2) were defined using data sourced from FishBase [58]. Data were collected for every species examined in the four studies, totalling 878 observations from 138 different species (Supporting Information S1 Table). Because the data were not normally distributed, a Chi-square (χ²) test with Bonferroni correction was used to compare the frequency of occurrence (FO, %) of microplastic across countries, while a Kruskal-Wallis (H) test with Dunn’s post-hoc analysis was applied to assess differences in microplastic load (mean ± standard error [SE], MP/individual). All statistical analyses were performed using Python (v3.11.11) in Google Colab [59]. The specific packages used include pandas, matplotlib, numpy, seaborn, scipy.stats, and scikit_posthocs.

**Table 2:** Definition of ecological trait categories and their groupings.

Ecological Trait	Definition and categories
Feeding Strategy	The method by which an organism captures and consumes food.
	**Ambush/biting/suction feeder:** Predators that wait and strike or engulf prey suddenly. **Filter/ram feeder:** Strain small particles including plankton and detritus. **Grazer:** Scrape or nibble on algae or plant material. **Pursuit feeder:** Actively chase and capture prey. **Surface feeder:** Feed on organisms or detritus at the water surface.
Feeding Mode	The primary diet of an organism.
	**Piscivore:** Primarily consume other fish. **Invertivore:** Specialize in feeding on invertebrates. **Omnivore:** Consume both plant material and animal prey. **Planktivore:** Specialize in feeding on plankton. **Herbivore:** Primarily consume algae and plant matter.
Habitat Use	The environment where an organism is commonly found.
	**Benthic-Demersal:** Live near or on the seafloor. **Coastal Pelagic/Lagoon:** Found in coastal waters, often in lagoons. **Open/Deep Water:** Inhabit deeper open ocean environments. **Reef Associated:** Associated with coral reefs and rocky structures.
Feeding Zone	The depth zone in which an organism primarily feeds.
	**Bottom feeder:** Feed at or near the seafloor. **Surface feeder:** Feed at or near the water’s surface. **Water column feeder:** Feed within the midwater column.

## Results

**Fig 2:**
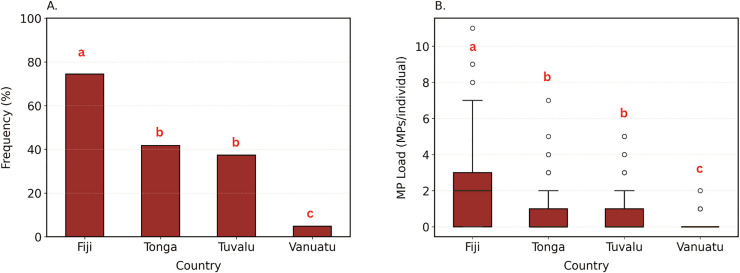
National-scale microplastic contamination patterns across four Pacific Island countries. (A) Overall frequency of occurrence (%) of microplastics in coastal fish species from Fiji, Tonga, Tuvalu, and Vanuatu, showing the proportion of contaminated individuals per country. (B) Mean microplastic load (MPs/individual ± SE) comparing average particle counts among countries. Error bars represent the standard error (SE). Letters (a–c) indicate statistical significance; bars sharing the same letter are not significantly different..

A total of 878 individual fish from a total of 138 species (56 Genera and 27 Families) were analysed across the four countries. Of these, microplastics were observed in 32.69% of fish GI tracts and on average microplastic load per individual fish was 0.76 ± 0.05 MP/individual. Across the four countries, both the FO and load varied significantly (respectively; χ² (3, 878) = 278.12, p < 0.001; H (3, 878) = 298.68, p < 0.001). Fiji exhibited the highest levels of microplastic contamination (Fig 2), with a FO of 74.46% and an average load of 2.17 ± 0.16 MP/individual, both significantly higher than the other countries (respectively; p < 0.001; p < 0.001). Tonga and Tuvalu showed intermediate levels, with FO values of 41.73% and 37.31%, respectively, and microplastic load values of 0.77 ± 0.10 and 0.72 ± 0.08 MP/individual, with no significant differences between them (respectively; p = 1.00; p = 1.00). Vanuatu had the lowest contamination levels, with an FO of 4.80% and a load of 0.05 ± 0.01 MP/individual, significantly lower than all other countries (p < 0.001 for both metrics).

Across all four countries fibers were the most common (~66–95%) microplastic type (Supporting Information S2 Table). Fragments represent the second most common type (~20–26%) in Fiji, Tonga, and Tuvalu, but were absent (0%) in Vanuatu. Films were present in samples from all four countries, but were the least prevalent (~5–14%). Size distribution patterns presented clear national differences. Fiji had a relatively even spread across size classes. Tonga had more medium-sized particles, Tuvalu showed a strong dominance of the smallest size class, and Vanuatu’s particles were mostly medium-sized. Polymer composition also showed country specific differences (Supporting Information S3 Table). In Fiji, polyethylene (PE), polypropylene (PP), polyethylene terephthalate (PET), and nylon were most common, each accounting for 16–20% of particles. Tonga’s microplastics were dominated by PP (34%) and PE (23%), Tuvalu’s by PP (43%) and nylon (20%), and Vanuatu’s by PE (38%), PP (23%), and polyvinyl acetate (23%). Minor polymers such as polyurethane, polystyrene, nitrile, and PVC occurred in small amounts (1–8%) in some countries.

**Fig 3:**
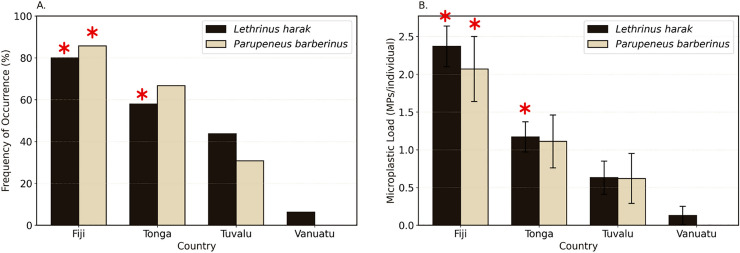
Microplastic contamination patterns in *Lethrinus harak* (thumbprint emperor) and *Parupeneus barberinus* (dash-and-dot goatfish) across Fiji, Tonga, Tuvalu, and Vanuatu. (A) Frequency of occurrence (%) of microplastic showing species-specific contamination rates by country. (B) Mean microplastic load (MPs/individual ± SE) comparing particle accumulation between species and locations. Error bars represent the standard error (SE), and asterisks indicate significant pairwise differences between countries.

Of the 138 fish species analyzed, 37 species were found across more than one country (Table 3). Microplastic FO varied considerably, ranging from 0% (e.g., *Acanthurus lineatus* in Tonga and *Acanthurus mata* in Fiji) to 100% (e.g., *Lutjanus gibbus* in Fiji and *Selar crumenophthalmus* in Tonga). Similarly, the mean microplastic load ranged from 0.0 ± 0.0 MP/individual to as high as 5.33 ± 0.88 MP/individual in *Selar crumenophthalmus* in Tonga. Two species, *Lethrinus harak* and *Parupeneus barberinus* were observed in all four countries (Fig 3). In *L. harak,* Fiji had the highest FO (80.00%), followed by Tonga (57.89%), Tuvalu (43.75%), and Vanuatu (6.25%). Significant differences were noted between Fiji and Tuvalu (χ² (3, 130) = 278.12, p < 0.001) and Tonga and Vanuatu (p = 0.017). Similarly, the microplastic load per fish also differed significantly by country (H (3,130) = 32.18, p < 0.001). Microplastic load in *L. harak,* was highest in Fiji (2.37 ± 0.27 MP/individual), significantly greater than Tonga (1.17 ± 0.20 MP/individual, p = 0.044), Tuvalu (0.63 ± 0.22 MP/individual, p = 0.007), and Vanuatu (0.13 ± 0.13 MP/individual, p < 0.001). Tonga’s load was also significantly higher than Vanuatu’s (p = 0.05). The difference in microplastic load within *L. harak* in Tonga and Tuvalu did not differ significantly (p = 1.000). For *Parupeneus barberinus,* the FO of microplastic was highest in Fiji (85.71%), followed by Tonga (66.67%) and Tuvalu (30.77%). No microplastics (0%) were observed in *P. barberinus* from Vanuatu, however only differences between Fiji and Tuvalu were significant (χ² (3,40) = 13.80, p = 0.03). Similarly, microplastic load varied across countries with Fiji recording a significantly higher load (2.07 ± 0.43 MP/individual) than Tuvalu (0.62 ± 0.33; H (3,40) = 12.58, p = 0.026). Tonga recorded a lower (1.11 ± 0.35 particles/fish) microplastic load than Fiji and higher load than Tuvalu, although the differences were not significant (H (3,40) = 12.58, p = 0.40).

**Table 3:** Frequency of occurrence, and microplastic load (mean ± SE) for species observed across countries. Empty cells reflect no data are available for that fish in the respective country.

Species	Fiji (FO% [Mean MP ± SE])	Tonga (FO% [Mean MP ± SE])	Tuvalu (FO% [Mean MP ± SE])	Vanuatu (FO% [Mean MP ± SE])
*Acanthurus lineatus*			100.00% [1.33 ± 0.33]	0.00% [0.00 ± 0.00]
*Acanthurus mata*		0.00% [0.00 ± 0.00]	100.00% [2.00 ± 0.00]	
*Acanthurus triostegus*			30.77% [0.92 ± 0.43]	0.00% [0.00 ± 0.00]
*Aphareus rutilans*			66.67% [2.00 ± 1.53]	0.00% [0.00 ± 0.00]
*Aprion virescens*		0.00% [0.0 ± 0.00]		0.00% [0.00 ± 0.00]
*Calotomus carolinus*		0.00% [0.00 ± 0.00]		0.00% [0.00 ± 0.00]
*Caranx melampygus*		50.00% [0.50 ± 0.50]		0.00% [0.00 ± 0.00]
*Cetoscarus ocellatus*	90.91% [1.82 ± 0.40]			0.00% [0.00 ± 0.00]
*Cheilinus trilobatus*		33.33% [0.67 ± 0.67]		0.00% [0.00 ± 0.00]
*Ellochelon vaigiensis*		33.33% [0.33 ± 0.33]		0.00% [0.00 ± 0.00]
*Epinephelus maculatus*		0.00% [0.00 ± 0.00]	15.38% [0.23 ± 0.17]	0.00% [0.00 ± 0.00]
*Hemiramphus far*	60.00% [1.45 ± 0.45]			20.59% [0.21 ± 0.07]
*Hipposcarus longiceps*		20.00% [0.20 ± 0.20]	50.00% [0.83 ± 0.48]	
*Kyphosus vaigiensis*	100.00% [4.0 ± 1.00]			0.00% [0.00 ± 0.00]
*Lethrinus erythracanthus*		40.00% [0.40 ± 0.25]	0.00% [0.00 ± 0.00]	
*Lethrinus harak*	80.00% [2.37 ± 0.27]	57.89% [1.17 ± 0.20]	43.75% [0.63 ± 0.22]	6.25% [0.13 ± 0.13]
*Lethrinus nebulosus*		38.46% [0.69 ± 0.33]		0.00% [0.00 ± 0.00]
*Lethrinus obsoletus*			12.50% [0.15 ± 0.15]	0.00% [0.00 ± 0.00]
*Lethrinus olivaceus*	50.0% [2.50 ± 2.50]		50.00% [1.50 ± 1.50]	0.00% [0.00 ± 0.00]
*Lutjanus fulvus*	100.00% [2.00 ± nan]	0.00% [0.00 ± 0.00]		0.00% [0.00 ± 0.00]
*Lutjanus gibbus*	100.0% [2.33 ± 0.67]	0.00% [0.00 ± 0.00]	0.00% [0.00 ± 0.00]	
*Monotaxis grandoculis*			20.00% [0.27 ± 0.15]	0.00% [0.00 ± 0.00]
*Mulloidichthys flavolineatus*			100.00% [2.67 ± 1.20]	50.00% [0.50 ± 0.50]
*Mulloidichthys vanicolensis*			75.00% [2.25 ± 1.03]	0.00% [0.00 ± 0.00]
*Myripristis berndti*	0.00% [0.00 ± 0.00]		33.33% [1.33 ± 1.33]	
*Naso unicornis*		0.00% [0.00 ± 0.00]	75.00% [1.50 ± 0.65]	
*Oxycheilinus digramma*		0.00% [0.00 ± 0.00]		0.00% [0.00 ± 0.00]
*Parupeneus barberinus*	85.71% [2.07 ± 0.43]	66.67% [1.11 ± 0.35]	30.77% [0.62 ± 0.33]	0.00% [0.00 ± 0.00]
*Parupeneus indicus*		0.00% [0.00 ± 0.00]		25.00% [0.25 ± 0.25]
*Parupeneus multifasciatus*			100.00% [1.00 ± nan]	0.00% [0.00 ± 0.00]
*Sargocentron spiniferum*		0.00% [0.00 ± 0.00]	75.00% [2.25 ± 1.11]	0.00% [0.00 ± 0.00]
*Scarus altipinnis*			0.00% [0.00 ± 0.00]	0.00% [0.00 ± 0.00]
*Scarus rivulatus*		50.00% [0.75 ± 0.31]		0.00% [0.00 ± 0.00]
*Selar crumenophthalmus*		100.00% [5.33 ± 0.88]		3.33% [0.03 ± 0.023]
*Siganus punctatus*		100.00% [1.00 ± nan]	0.00% [0.00 ± 0.00]	
*Sphyraena barracuda*	55.00% [1.00 ± 0.31]			0.00% [0.00 ± 0.00]
*Terapon jarbua*	100.0% [3.50 ± 0.65]			0.00% [0.00 ± 0.00]

**Fig 4:**
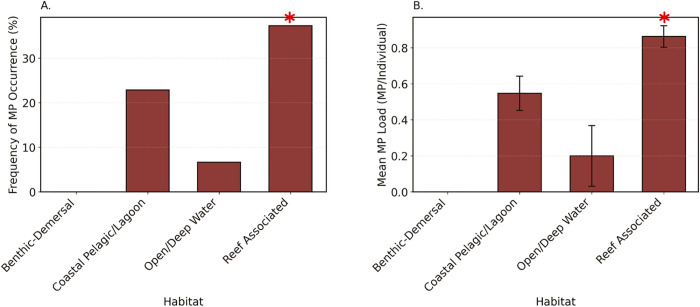
Microplastic contamination in coastal subsistence fish from Fiji, Tonga, Tuvalu, and Vanuatu, stratified by habitat use. (A) Frequency of microplastic occurrence (%), representing the proportion of individuals with detectable microplastics per habitat type. (B) Mean microplastic load (MPs/individual ± SE) across habitats. Error bars represent the standard error (SE), and asterisks indicate significant pairwise differences between countries.

The presence of microplastics varied significantly among fish occupying different habitats in both FO (χ² (3, 878) = 25.48, p < 0.001), and load (H (3, 878) = 25.04, p < 0.001; Fig 4). Fish from reef-associated habitats exhibited significantly greater FO (37.26%, p = 0.001) and mean microplastics load (0.86 ± 0.06 MP/individual, p < 0.001) compared to those from coastal pelagic/lagoon habitats (FO: 23.29%, MP load: 0.54 ± 0.09 MP/individual) and open/deep water zones (FO: 6.67%, MP load: 0.20 ± 0.17 MP/individual). Notably, no microplastics were detected in fish from benthic-demersal habitats. Coastal pelagic/lagoon and open/deep water habitats did not differ significantly from each other in FO (p = 0.39) or load (p = 0.54).

**Fig 5:**
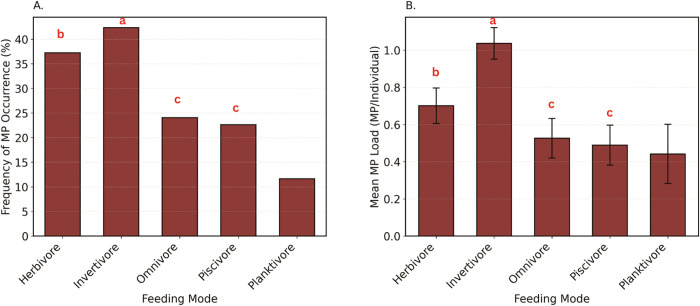
Microplastic contamination in coastal subsistence fish from Fiji, Tonga, Tuvalu, and Vanuatu, stratified by feeding mode. (A) Frequency of microplastic occurrence (%), representing the proportion of individuals with detectable microplastics per feeding mode. (B) Mean microplastic load (MPs/individual ± SE) across feeding modes. Error bars represent the standard error (SE), and letters (a–c) above the bars indicate significant pairwise differences among feeding modes.

Contamination varied significantly across feeding modes (Fig 5) in both FO (χ² (4, 878) = 45.71, p < 0.001) and microplastic load (H (4,878) = 44.40, p < 0.001). Invertivores (FO: 42.34%; MP load: 1.04 ± 0.08 MP/individual) showed the highest contamination, statistically exceeding omnivores (FO: 24.06%, p = 0.003; MP load: 0.53 ± 0.11 MP/individual, p < 0.001), piscivores (FO: 22.63%, p < 0.001; MP load: 0.49 ± 0.11 MP/individual, p < 0.001), and planktivores (FO: 11.63%, p < 0.001; MP load: 0.44 ± 0.16 MP/individual; p < 0.001), but not herbivores (FO: 37.23%, p = 1.000; MP load: 0.70 ± 0.10 MP/individual; p = 1.000). Herbivores were statistically higher than planktivores (FO: p < 0.001; MP load: p = 0.005) but did not statistically differ from omnivores or piscivores (FO: p = 0.27 and 0.12; MP load: p = 0.35 and 0.15). Omnivores, piscivores, and planktivores did not statistically differ from each other in FO (p ≥ 0.35) and in microplastic load (all p = 1.00).

**Fig 6:**
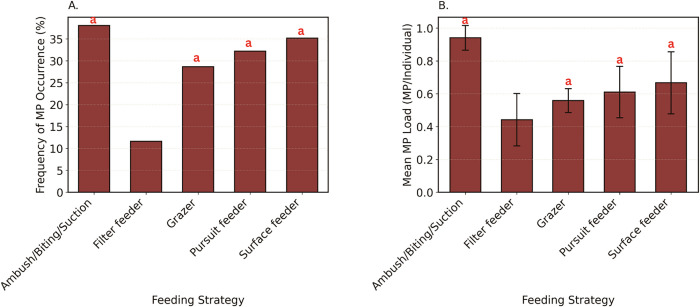
Microplastic contamination in coastal subsistence fish from Fiji, Tonga, Tuvalu, and Vanuatu, stratified by feeding strategy. (A) Frequency of microplastic occurrence (%), showing the proportion of contaminated individuals per feeding strategy group. (B) Mean microplastic load (MPs/individual ± SE), demonstrating particle accumulation across strategies. Error bars represent the standard error (SE), and letters (a–c) above the bars indicate significant pairwise differences among feeding modes.

The presence of microplastics varied significantly among fish with different feeding strategies (Fig 6) for both FO (χ² (4,878) = 25.22, p < 0.001) and load (H (4,878) = 23.97, p < 0.001). Ambush/biting/suction feeders had the highest contamination (FO: 38.05%; MP load: 0.94 ± 0.07 MP/individual), followed by surface feeders (FO: 35.19%, MP load: 0.67 ± 0.19 MP/individual), pursuit feeders (FO: 32.20%, MP load: 0.61 ± 0.16 MP/individual), and grazers (FO: 28.64%, MP load: 0.56 ± 0.07 MP/individual). These four groups did not differ significantly from each other in either FO or microplastic load (all p > 0.05), but all showed significantly greater contamination compared to filter feeders (FO: 11.63%, p < 0.001; MP load: 0.44 ± 0.16 MP/individual, p < 0.001).

**Fig 7:**
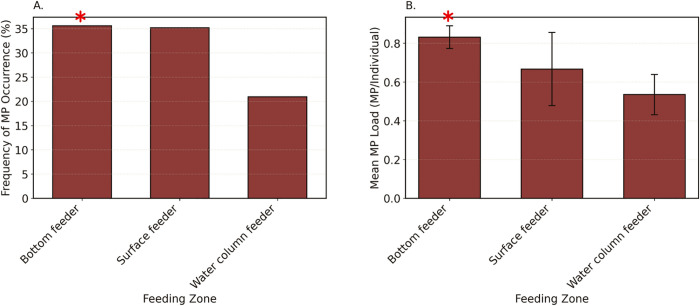
Microplastic contamination in coastal subsistence fish from Fiji, Tonga, Tuvalu, and Vanuatu, stratified by feeding zone. (A) Frequency of microplastic occurrence (%), showing the proportion of contaminated individuals per feeding zone. (B) Mean microplastic load (MPs/individual ± SE), demonstrating particle accumulation across zones. Error bars represent the standard error (SE), and asterisks indicate significant pairwise differences between feeding zones.

Along the feeding zones (Fig 7; across the water column), MP contamination varied significantly for both FO (χ² (2,878) = 13.44, p = 0.001) and load (H (2, 878) = 12.70, p = 0.001). Bottom-feeding fish (FO: 35.58%; MP load: 0.83 ± 0.06 MP/individual) showed the highest contamination levels, significantly exceeding water column feeders (FO: 20.93%, p = 0.001; MP load: 0.53 ± 0.10 MP/individual, p = 0.001). Surface feeders (FO: 35.19%; MP load: 0.67 ± 0.19 MP/individual) exhibited intermediate contamination that did not differ significantly (all p > 0.1) from either bottom feeders or water column feeders.

## Discussion

This study provides the first regionally coordinated synthesis of microplastic contamination in coastal fish across the PICTs, encompassing Fiji, Tonga, Tuvalu, and Vanuatu. While prior studies have been conducted in some of these countries, they were largely isolated and lacked standardized methods, limiting their comparability. Analysis of 878 individuals from 138 species revealed widespread microplastic presence, with 32.69% of fish containing microplastic in their gastrointestinal tracts, and an overall mean load of 0.76 ± 0.05 MP per fish. However, contamination patterns varied markedly across countries, habitats, and feeding groups. Fiji showed significantly higher levels of microplastic occurrence and particle loads compared to the other countries, and fish from reef-associated habitats and bottom feeders were particularly affected. Feeding behavior also influenced exposure, with invertivorous and herbivorous fish, as well as ambush/biting feeders, surface feeders, pursuit feeders, and grazers generally exhibited higher contamination than planktivorous and filter feeders. These findings highlight the spatial and ecological complexity of microplastic exposure among Pacific Island fish assemblages, with important implications for food security and ecosystem health.

The overall microplastic frequency occurrence and loads were lower than the global average (49% frequency of occurrence; 3.5 MP/individual) presented in Wootton et al. [11], but are consistent with tropical fish studies from similar systems [60]. Fiji emerged as an exception, exceeding global frequency rates (74.5%) though not load averages (2.17 ± 1.2 MPs/individual). This pattern persists even when compared to urban-focused studies [15], suggesting Fiji’s contamination reflects systemic factors such as population density, riverine inputs, and coastal development rather than localized urban pollution. The results highlight the ubiquity of microplastics in the coastal environment of Fiji compared to other studied PICTs. While direct overall comparisons are compounded by the different species assemblages in each country, comparisons of two species that were consistently sampled across the four countries (*L. harak* and *P. barberinus*) reflect the same pattern. The higher microplastic occurrence and loads in Fiji may be explained by the larger population and land area, and greater proximity to pollution sources (more rivers, coastal development), or may suggest less effective waste management practices in Fiji than other PICTs [61]. Vanuatu’s paradoxically low fish contamination (despite high beach litter near urban centers, [62]) suggests that dispersal from localized pollution input is not spread through rural coastal systems, possibly due to oceanographic currents, waste management buffers or differential bioaccumulation pathways. The moderate levels of microplastics present in Tonga and Tuvalu are surprising given their small populations and highlight the need to conduct further assessments in these countries given the heavy reliance on fish for food security. The data used in this study were the first reports of microplastics from Tuvalu [51], and on microplastics in fish from Tonga [50] where previous research has revealed moderate levels in coastal sediments [29], and waters [28].

Contamination levels varied significantly across species, with some species (e.g., *Lutjanus gibbus* in Fiji, *Selar crumenophthalmus* in Tonga) always having microplastics, and others (e.g., *Acanthurus lineatus* in Tonga, *Acanthurus mata* in Fiji) never having them. Such differences within countries suggest that feeding strategies and habitat strongly influence exposure to microplastics. Further addressing these differences can help us better understand contamination pathways, to understand potential implications for human health (based on consumption of different species) and to identify suitable indicator species for future research to focus on. When grouping all data, we found clear evidence of higher contamination rates in reef-associated fish, in those that feed from the benthic environment, and that are invertivores, (particularly when they are ambush feeders), or herbivores. This highlights that indirect consumption via sediments (for invertivores) or algae (herbivores), or biomagnification (for invertivores), may be key predictors of microplastic exposure. One example of such a fish is *L. harak* (a benthic-feeding invertivore), which has been the focus of other pollution studies [15,18], reinforcing the suitability of this species as a suitable indicator of pollution. This species is also one of the most common fish in the market in Fiji and is critical for commercial and subsistence fisheries [63], which highlights the concern that 80% of the individuals in Fiji were found to contain microplastics.

The dominance of fibers across all four countries in this study aligns with broader observations globally, as highlighted by Sacco et al. [13] and Oza et al. [64], who identify fibers as the most prevalent form of microplastics ingested by fish in natural environments. In our dataset, fibers accounted for over two-thirds of particles in Fiji, Tonga, and Tuvalu, and approximately 95% in Vanuatu, substantially higher than fragment and film proportions. This pattern is likely driven by the ubiquity of synthetic fibers shed from textiles, ropes, moorings and fishing gear and their visual and structural similarity to natural prey like zooplankton [65].

Across the four PICTs, there was variation in overall polymer profiles but clear similarity in the most dominant types. When compared to other studies from within the region, our findings are broadly consistent, with polyethylene, polypropylene and polyethylene terephthalate repeatedly reported as dominant polymers across different coastal constituents, including surface waters, water column, sediments, and marine biota [14,24,27–29]. Variations in the relative abundance of specific polymers between countries may reflect differences in local waste management capacity, population density, fishing gear and intensity, and the types of imported consumer goods and packaging in circulation. Nevertheless, the consistency in dominant polymer types, particularly the ubiquity of polyethylene and polypropylene, underscores their persistence in marine systems and reinforces the need for regionally coordinated monitoring to better understand their sources, pathways, and potential risks. From a hazard perspective, polyethylene and polypropylene are low-density, hydrophobic polymers that can adsorb and transport persistent organic pollutants such as PCBs and PAHs [66]. Polyethylene terephthalate and nylon, being denser, are more likely to sink and enter benthic food webs, where ingestion by demersal species could facilitate trophic transfer. Although hazard rankings place many of these polymers in lower toxicity categories, their widespread chronic presence and pollutant binding capacity still pose ecological risks, particularly in island ecosystems where waste management infrastructure is limited [67,68].

This study documents widespread microplastic contamination in Pacific Island coastal fisheries, with prevalence and loads varying significantly across species, habitats, and nations. The observed patterns reflect distinct environmental and anthropogenic drivers, including population density, waste management systems, and hydrological connectivity, that collectively influence exposure risks. Fiji’s elevated contamination levels (74.5% frequency) highlight the pressures of rapid development, while Vanuatu’s comparatively low microplastic loads suggest the mitigating role of oceanographic dynamics and policy interventions. The ecological insights into disproportionate microplastic ingestion by fish with certain ecological traits are critical for policy formulation [3,13], as they enable the identification of vulnerable marine food web components and the human communities dependent on these fisheries, directly linking microplastic pollution to food security and public health. Effective policy responses must prioritize both the protection of high-risk species and systemic reductions in plastic pollution at its source. This necessitates a shift away from superficial clean-up efforts and inadequate downstream solutions, such as recycling schemes or plastic credit initiatives, which remain ill-suited to the geographical and economic realities of PICTs [47,69]. Instead, upstream interventions including production controls, import regulations, and enhanced waste management systems are essential to prevent plastic from entering marine environments.

The policy and management implications of this research underscore the need for robust national and regional commitments. At the national level, PICTs must adopt stringent regulations to curtail plastic consumption and leakage, including comprehensive bans on unnecessary single-use plastics, investments in publicly owned waste infrastructure, and restrictions on the import of unmanageable plastic products [69,70]. Scientific findings such as those presented here should directly inform policy priorities, guiding resource allocation and enforcement strategies [32]. The notably lower microplastic levels in Vanuatu, though requiring further investigation to account for site-specific variables, may offer valuable insights into the efficacy of proactive policies, serving as a potential model for regional adaptation if empirically validated [61]. Regionally, PICTs must strengthen collaborative efforts through platforms such as the Pacific Regional Environment Programme (SPREP), leveraging collective political influence to harmonize policies and resist the influx of harmful plastics. Given the transboundary nature of marine pollution, regional cooperation is scientifically and strategically imperative [71].

A legally binding Global Plastics Treaty is critical to reinforce these national and regional efforts. The ongoing negotiations under the Intergovernmental Negotiating Committee represent a pivotal opportunity to address plastic pollution at its source [72]. The findings of this study provide compelling evidence for PICTs to advocate for a treaty mandating global reductions in primary plastic production [73], the phase-out of hazardous plastic additives [74], and enhanced transparency across the plastic lifecycle [75]. Additionally, the treaty must include financial and technical mechanisms to support SIDS in implementation [76]. Without such a framework, even the most rigorous regional measures risk being overwhelmed by the scale of global plastic production [61]. The scientific data from the Pacific should thus inform global policy to ensure systemic change, rather than perpetuating a pollution burden disproportionately borne by nations least responsible for its creation.

Methodological consistency remains a challenge in microplastic research, as highlighted by Wootton et al. [11], underscoring the value of standardized approaches like those employed in this study. However, limitations persist, including the need for larger species-specific sample sizes to improve comparative analyses and elucidate trophic transfer pathways. Future research should also investigate the translocation of nanoplastics, chemical additives, and adsorbed pollutants into edible tissues to better assess human health risks associated with microplastic-contaminated seafood. Addressing these gaps will refine risk assessments and strengthen evidence-based policy interventions.

## Conclusion and future perspectives

This study provides a regionally coordinated synthesis of microplastic contamination in coastal fish across four PICTs revealing both alarming trends and unique regional dynamics. While overall contamination levels (32.7% prevalence; 0.76 ± 0.05 MPs/individual) were lower than global averages, Fiji had notably higher contamination levels (74.5% prevalence) with microplastic frequencies comparable to global averages. The consistent pattern of high contamination in reef-associated species across borders confirms ecological traits as key exposure predictors, while national disparities highlight the failure of current waste management systems, or lack thereof to protect even remote island ecosystems. These findings highlight a key environmental challenge for Pacific nations, where heavy reliance on coastal fisheries transforms ecological contamination into immediate food security and health risks. The dominance of polymers associated with fishing gear and ropes (polyethylene/polypropylene/nylon) and fibers (65–95% of particles) suggests that marine-based pollution pathways should also be considered under conventional land-focused waste policies.

To advance progress, we recommend three interconnected pathways to guide action: First, comprehensive exposure assessments to trace microplastics from fishery to human consumption, analysing how fishing gear types, post-harvest processing methods, and traditional preparation techniques influence contamination levels in edible portions, with special attention to vulnerable consumer groups. Second, community-led monitoring programs must integrate scientific methods with traditional ecological knowledge to map pollution sources and identify locally appropriate solutions, from gear modifications to seasonal harvesting guidelines. Third, these initiatives must directly inform policy through adaptive tools like dynamic exposure risk indices, while ensuring Pacific data and perspectives shape global treaty negotiations, challenging assumptions that global pollution models adequately represent SIDS vulnerabilities. Successful mitigation strategies would recognize that plastic contamination is as much a social challenge as an ecological one. Centering Pacific knowledge systems and lived experiences must be at the heart of both research and solutions is essential. From understanding how local practices affect microplastic exposure to co-designing risk communication with fishing communities, this approach enables interventions that are both scientifically rigorous and culturally grounded.

## Supporting information

S1 Table Morphometric and ecological characteristics of sampled fish species across study countries, including total length, fork length, total weight, trophic level, feeding type, feeding strategy, habitat preference, and water column position. Data on trophic levels and associated ecological characteristics was accessed from Fishbase on the 18 of August 2025.
(CSV)

S2 Table Microplastic (MP) characteristics by country, showing form type and size distribution across Fiji, Tonga, Tuvalu, and Vanuatu. (XLSX)

S3 Table Polymer composition (%) of microplastics (MPs) analyzed in subsamples from Fiji, Tonga, Tuvalu, and Vanuatu. Empty cells reflect no occurrence of that polymer type in analyzed subsamples.
(XLSX)
